# Gemcitabine/cisplatin induction chemotherapy before concurrent chemotherapy and intensity-modulated radiotherapy improves outcomes for locoregionally advanced nasopharyngeal carcinoma

**DOI:** 10.18632/oncotarget.18245

**Published:** 2017-05-27

**Authors:** Wang Fangzheng, Sun Quanquan, Jiang Chuner, Wang Lei, Yan Fengqin, Ye Zhimin, Liu Tongxin, Xu Min, Wu Peng, Jiang Haitao, Rihito Aizawa, Masoto Sakamoto, Wang Yuezhen, Fu Zhenfu

**Affiliations:** ^1^ Department of Radiation Oncology, Zhejiang Cancer Hospital, Zhejiang Hangzhou, 310022, People’s Republic of China; ^2^ Zhejiang Key Laboratory of Radiation Oncology, Zhejiang Hangzhou, 310022, People’s Republic of China; ^3^ Department of Radiology, Japanese Red Cross Fukui Hospital, Fukui, 918-8501, Japan; ^4^ Department of Breast Tumor Surgery, Zhejiang Cancer Hospital, Zhejiang Hangzhou, 310022, People’s Republic of China; ^5^ Department of Physics, Zhejiang Cancer Hospital, Zhejiang Hangzhou, 310022, People’s Republic of China; ^6^ Department of Pathology, Zhejiang Cancer Hospital, Zhejiang Hangzhou, 310022, People’s Republic of China; ^7^ Department of Radiology, Zhejiang Cancer Hospital, Zhejiang Hangzhou, 310022, People’s Republic of China; ^8^ Department of Radiation Oncology and Image-applied Therapy, Graduate School of Medicine, Kyoto University, Kyoto, 606-8507, Japan

**Keywords:** nasopharyngeal carcinoma, induction chemotherapy, intensity-modulated radiotherapy, concurrent chemotherapy, toxicity

## Abstract

Addition of induction chemotherapy (IC) to concurrent chemoradiotherapy (CC) is an encouraging first-line treatment strategy for patients with locoregionally advanced nasopharyngeal carcinoma (NPC). We evaluated the clinical efficacy and toxicity of addition of gemcitabine plus cisplatin (GP) IC to intensity-modulated radiotherapy (IMRT) and CC for patients with locoregionally advanced NPC. At a median follow-up duration of 48 months (10–59 months), 4-year local relapse-free survival (LRFS) was 86.9%, regional relapse-free survival (RRFS) was 90.6%, distant metastasis-free survival (DMFS) was 79.8%, progression-free survival (PFS) was 77.0%, and overall survival (OS) was 81.9%. Univariate analysis revealed that *T* stage, *N* stage, clinical stage, and CC correlated with OS, while *N* stage and clinical stage correlated with PFS. In multivariate analysis, T4 was a prognostic indicator of poor OS and PFS, and N3 was a prognostic indicator of poor OS. Having received ≥ 2 cycles of IC was prognostic of better RRFS. During IC, grade 3–4 thrombocytopenia occurred in 10 patients, and grade 3–4 leukocytopenia was observed in 16 patients. Two patients developed mild liver dysfunction. These findings indicate that GP-based IC followed by CC has promising efficacy with acceptable toxicities.

## INTRODUCTION

NPC is endemic in Singapore, Malaysia, and Southern China, with an incidence of 15–50 cases per 100,000 [1]. Because of the anatomical location of the nasopharynx and high sensitivity to irradiation, RT is regarded as a prime treatment strategy for non-disseminated NPC. The survival outcomes of NPC patients have improved continually due to advances in radiological techniques, extensive application of IMRT, and the addition of CC [1, 2]. Although the 5-year OS rates are 90–100% for stage I–II and 60–85% for stage III–IVB, distant metastasis remains the primary source of treatment failure for NPC patients [3, 4]. Unfortunately, more than 70% of patients are diagnosed with NPC when it is already locoregionally advanced [5]. Adjuvant chemotherapy (AC) has failed to improve survival outcomes for these patients due to the low completion rate for a full course of three cycles [6]. IC can improve patients’ tolerability, eradicate micrometastases, and protect normal tissue due to the reduction of tumor compared with AC. Thence, IC followed by concurrent chemoradiotherapy (CCRT) appears to be an encouraging option to further improve survival outcomes of patients with locoregionally advanced NPC and is recommended by the 2014 National Comprehensive Cancer Network (NCCN) guidelines [7].

A recent Phase 3 multi-center, randomized trial published in Lancet Oncology indicated that addition of docetaxel, cisplatin, and 5-flurouracil (TPF) to CCRT significantly improved OS, failure-free survival, and DMFS rates of patients with locoregionally advanced NPC [8]. Kong L et al. also recently demonstrated that addition of TPF-based IC to CCRT increased survival outcomes of locoregionally advanced NPC patients in comparison with historical data [9].

The combination of gemcitabine with cisplatin (GP) confers synergistic cytotoxic effects *in vitro* [10]. The results from a multi-center, randomized, phase 3 trial established a GP regimen as the first-line treatment for patients with recurrent or metastatic NPC because it improved PFS and OS [11]. Pan JJ et al. showed that a GP regimen prolonged OS and had a tendency to increase DMFS [12]. Shi M et al. recently indicated that in subgroup analysis, a GP regimen significantly expanded OS compared with TP or FP [13].

Because the number of patients receiving GP-based IC in the above two studies was small, it remains uncertain whether GP is an effective and safe regimen for locoregionally advanced NPC. We conducted a phase II trial to evaluate the clinical efficacy and toxicity of GP regimen as a first-line IC modality before CCRT for locoregionally advanced NPC.

## RESULTS

### Basic characteristics of patients and treatment compliance

Between January 2012 and January 2014, a total of 74 patients newly diagnosed with locoregionally advanced NPC were enrolled. Basic characteristics of patients are summarized in Table 1. The median age was 55 years (range: 18–70 years). All patients completed a full course of definitive IMRT and received ≥ 1 cycles of IC. Among these patients, 56 (75.7%) were administered CC, and 47 (63.5%) received AC (Table 2).

**Table 1:** Basic characteristics of 74 patients with locoregionally advanced NPC

Characteristic	Patients	
	No	%
Gender		
Male	53	71.6
Female	21	28.4
Age (years)		
Range	18–70	
Median	55	
< 50	30	40.5
≥ 50	44	59.5
WHO pathology		
Type I	3	4.1
Type II	2	2.7
Type III	69	93.2
ECOG performance status		
0	64	86.5
1	10	13.5
T stage ^*^		
T1	1	1.4
T2	28	37.8
T3	30	40.5
T4	15	20.3
N stage ^*^		
N0	1	1.4
N1	11	14.9
N2	55	74.3
N3	7	9.4
Clinical stage ^*^		
III	53	71.6
IV	21	28.4
Comorbidity		
No	51	68.9
Yes	23	31.1

Abbreviations: WHO World Health Organization, ECOG Eastern Cooperative Oncology Group, ^*^ The 7th AJCC/UICC staging system.

**Table 2:** Treatment compliance in 74 patients with locoregionally advanced NPC

Treatment compliance	*N* (%)
Cycle of IC	
1	9 (12.2)
2	58 (78.4)
3	7 (9.4)
Cycle of CC	
No	18 (24.3)
1	36 (48.6)
2	20 (27.1)
AC	
No	27 (36.5)
Yes	47 (63.5)
AC regimens	
GP	25 (53.2)
FP	22 (46.8)

### Disease response

IC achieved complete remission (CR) in 19 patients (25.6%), partial remission (PR) in 52 patients (70.3%), and stable disease (SD) in 3 patients (4.1%) for lesions of the nasopharynx. CR, PR, and SD rates of cervical lymph nodes for 73 patients with neck metastatic lymph nodes were 41.1% (30/73), 56.2% (41/73), and 2.7% (2/73), respectively. Among 9 patients who received one cycle of IC, 7 achieved PR and 2 achieved SD for nasopharyngeal tumors; 6 achieved PR and 2 achieved SD for neck lymph nodes. Of 65 patients who received 2–3 cycles of IC, CR, PR and SD rates were 29.2% (19/65), 69.2% (45/65), and 1.6% (1/65), respectively for nasopharyngeal tumor and 46.2% (30/65), 53.8% (35/60), and 0 (0/60), respectively, for neck lymph node.

At the end of IMRT, CR rates of nasopharyngeal tumor and neck metastatic lymph nodes were 97.3% and 98.6%, respectively.

### Treatment efficacy

**Figure 1:**
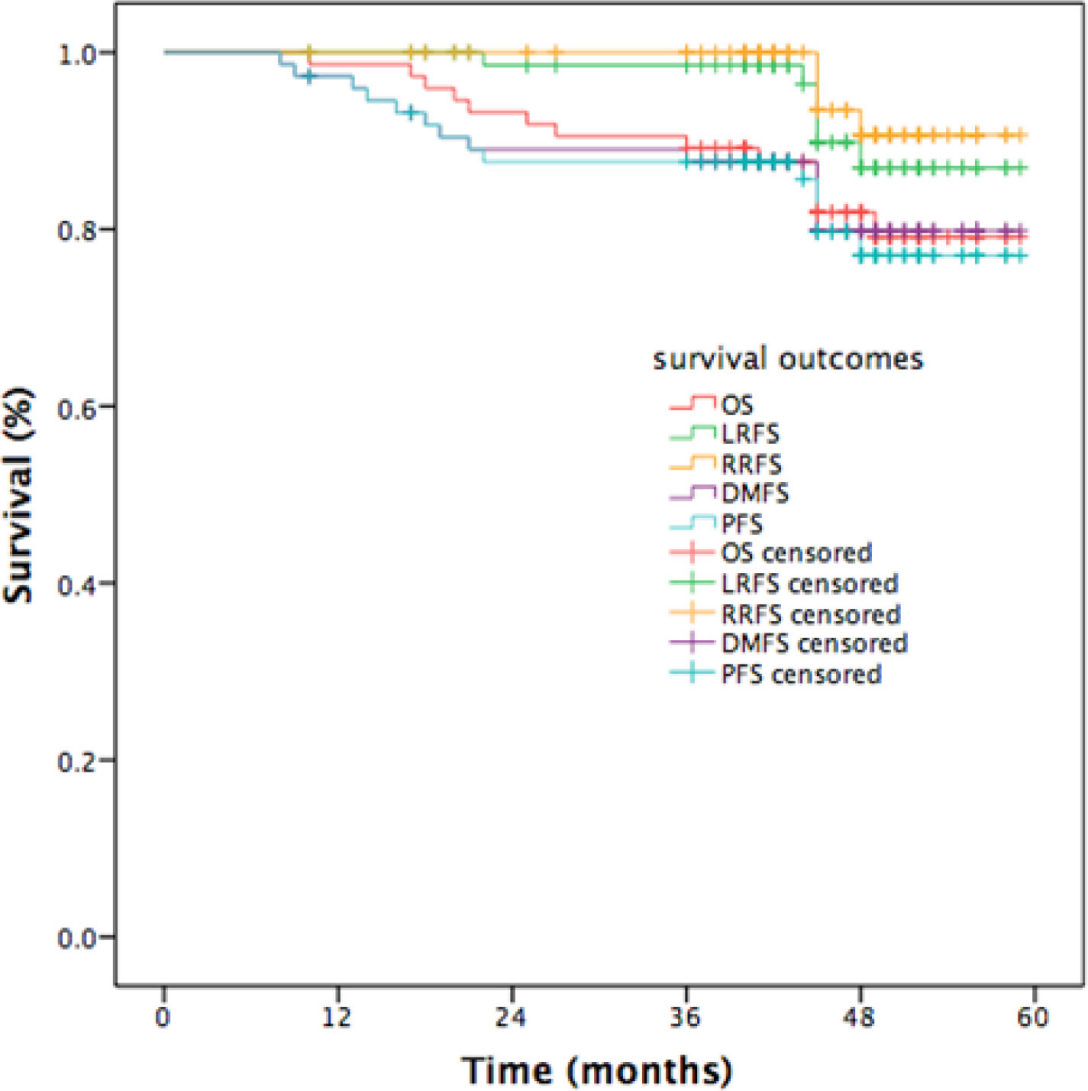
Kaplan-Meier curves of survival outcomes in patients with NPC

**Figure 2:**
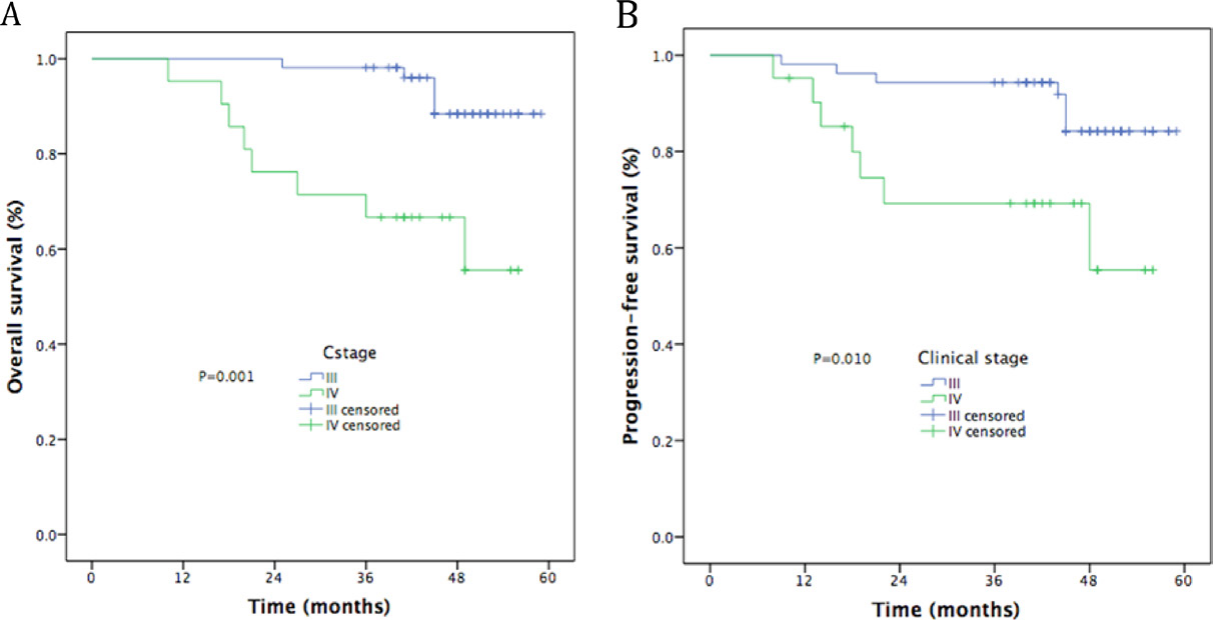
Kaplan-Meier curves of survival outcomes by clinical stage (**A**) Overall survival; (**B**) Progression-free survival.

**Figure 3:**
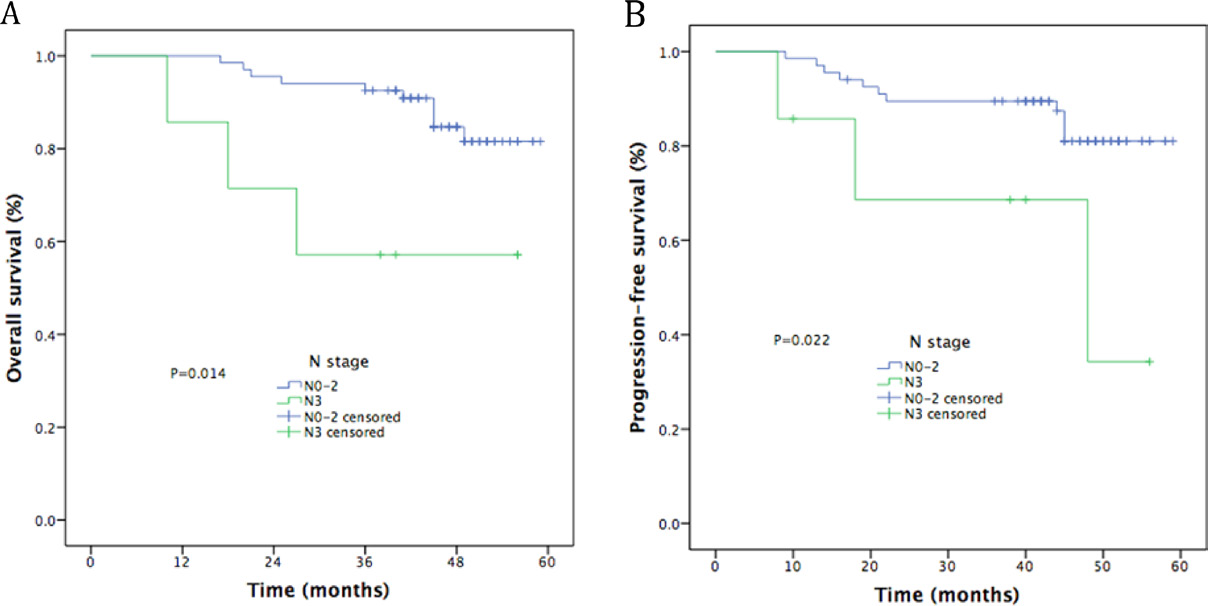
Kaplan-Meier curves of survival outcomes for N stage (**A**) Overall survival; (**B**) Progression-free survival.

**Figure 4:**
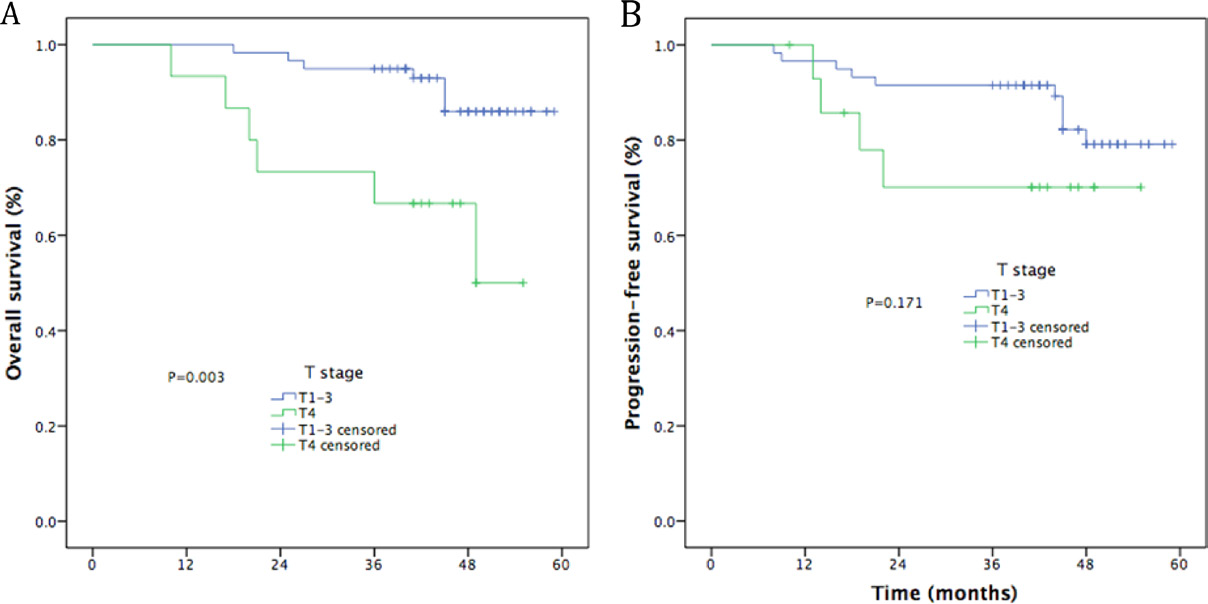
Kaplan-Meier curves of survival outcomes for T stage (**A**) Overall survival; (**B**) Progression-free survival.

**Figure 5:**
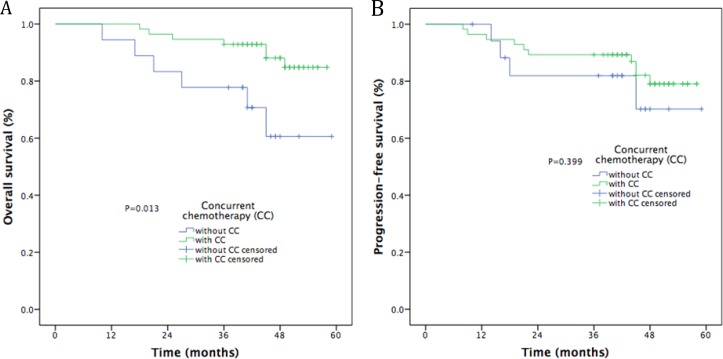
Kaplan-Meier curves of survival outcomes in patients treated with or without concurrent chemotherapy (**A**) Overall survival; (**B**) Progression-free survival.

The median follow-up time was 48 months (range, 10–59). The estimated 4-year OS, LRFS, RRFS, DMFS, and PFS rates were 86.9%, 90.6%, 79.8%, 77.0%, and 81.9%, respectively (Figure 1). The 4-year OS and PFS rates were 88.4% and 55.6%, for patients with stage III (*P* = 0.001, Figure 2A) and 84.2% and 55.4% for patients with stage IV (*P* = 0.010, Figure 2B). Moreover, the 4-year OS and PFS rates were 84.7% and 57.1% for patients with stage N0-2 (*P* = 0.014, Figure 3A) and 81.0% and 34.3% for patients with stage N3 (*P* = 0.022, Figure 3B). Patients with stage T4 had poorer OS rates than those with stage T1-3 (50.0% vs. 86.0%, *P* = 0.003, Figure 4A). Although the 4-year PFS rate of patients with stage T1-3 was higher than that of patients with stage T4, there was no significantly statistical difference (79.1% vs. 70.1%, *P* = 0.171, Figure 4B). The 4-year OS rate of patients treated with CC was higher than that of the patients who did not receive CC (88.1% vs. 60.6%, *P* = 0.036, Figure 5A). Patients treated with CC had better PFS rates than those treated without CC, but the difference is not statistically significant (82.1% vs. 70.2%, *P* = 0.399, Figure 5B).

### The mode of treatment failure

**Figure 6:**
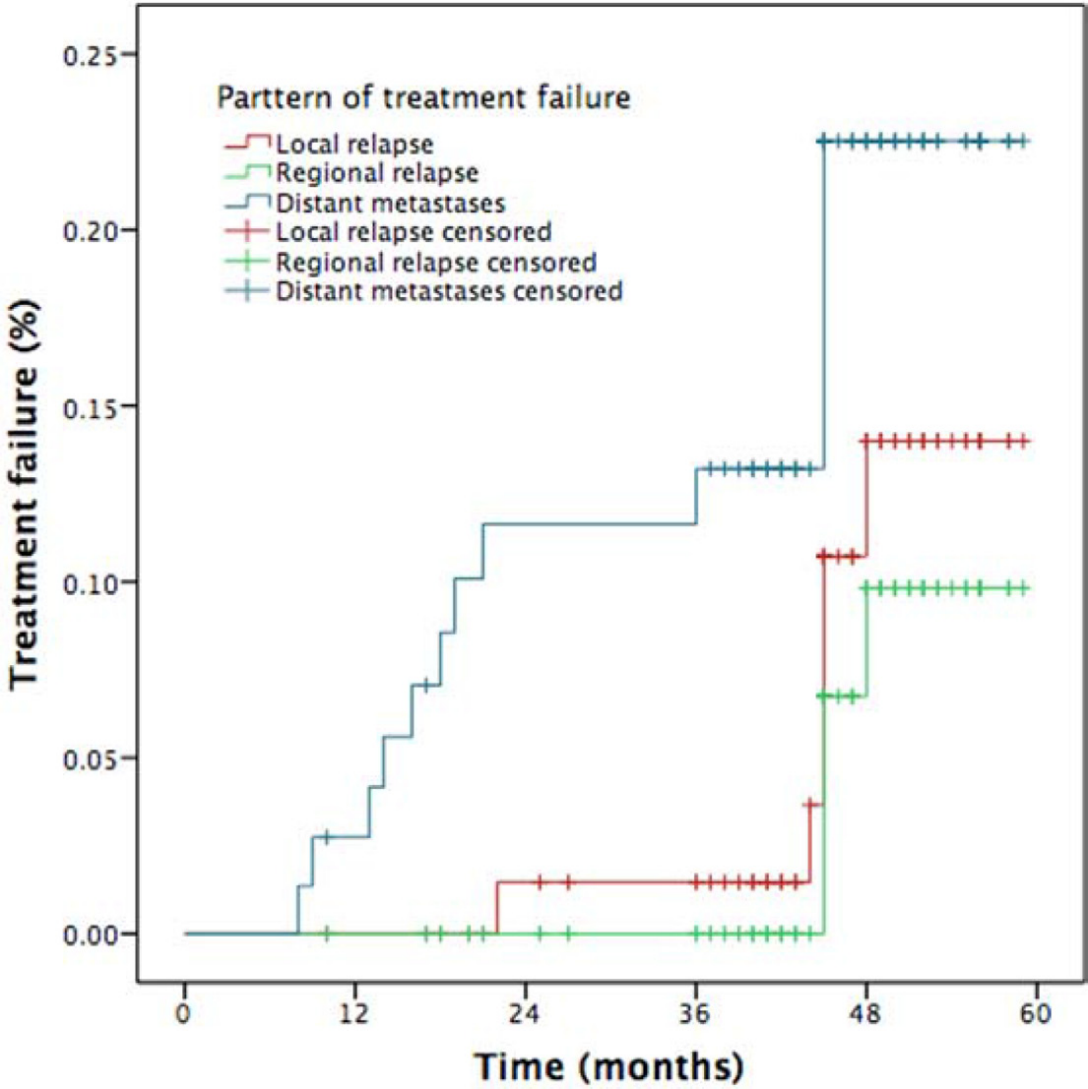
Patterns of treatment failure for NPC patients

Altogether, 15 patients developed treatment failure by the last follow-up: local relapse was found in only one patient; loco-regional relapse occurred in 1 patient; loco-regional relapse and distant metastases were found in three patients, and 10 patients experienced only distant failure. Among the metastatic sites, 5 cases occurred in lung, 4 in bone, 2 in liver, and 2 in multiple locations. Patterns of treatment failure in NPC patients are listed in Figure 6. Distant metastasis was the main cause of failure (*P* = 0.042).

### Analysis of prognostic factors

We evaluated several potential prognostic factors including patient age, gender, comorbidities, *T* stage, *N* stage, clinical stage, IC cycle, CC, and AC. Univariate analysis revealed that clinical stage and CCRT regimens were significant prognostic factors for OS, while clinical stage was a significant prognostic factor for PFS (Table 3). In multivariate analysis, N3 was a prognostic factor for poorer OS and PFS, T4 for poorer OS, and receiving only 1 cycle of IC for poorer RRFS (Table 4).

**Table 3:** Univariate analysis of prognostic factors for OS and PFS in LA NPC patients

Characteristic	*N*	4-year OS (%)	*P*	4-year PFS (%)	*P*
Gender			0.794		0.874
Male	53	84.2		78.0	
Female	21	74.7		74.0	
Age (years)			0.974		0.880
< 50	30	82.7		83.0	
≥ 50	44	81.8		73.9	
T stage ^*^			0.103		0.924
T1-2	29	88.7		76.8	
T3-4	45	77.1		88.4	
N stage ^*^			0.301		0.234
N0–1	12	90.9		90.9	
N2–3	62	80.4		74.4	
Clinical stage ^*^			0.001		0.010
III	53	88.4		84.2	
IV	21	55.6		55.4	
Comorbidity			0.463		0.321
No	51	81.2		74.1	
Yes	23	83.7		83.7	
Cycle of IC			0.927		0.591
1	9	74.1		49.4	
2	58	82.8		80.0	
3	7	85.7		85.7	
CC			0.013		0.399
No	18	60.6		70.2	
Yes	56	88.1		79.1	
AC			0.149		0.229
No	27	76.7		65.6	
Yes	47	84.9		83.0	

^*^ The 7th AJCC/UICC staging system.

**Table 4:** Multivariate analysis of prognostic factors in LA NPC patients

	Characteristic	HR	95% CI	*P*-value
OS	T1-3 vs. T4^*^	0.150	0.044–0.508	0.002
	N0-2 vs. N3^*^	0.121	0.028–0.520	0.005
PFS	N0-2 vs. N3^*^	0.250	0.069–0.902	0.034
LRFS	-	-	-	-
RRFS	1 vs. 2-3 cycles IC	7.374	1.031–52.743	0.047
DMFS	-	-	-	-

Abbreviations: OS: overall survival; PFS: progression-free survival; LRFS: local recurrence-free survival; RRFS: regional recurrence-free survival; DMFS: distant metastasis-free survival; IC induction chemotherapy; ^*^The 7th AJCC/UICC staging system.

### Acute side effects

The most common treatment-related acute toxicities are listed in Table 5. During the course of IC, the following grade 3–4 acute hematologic toxicities occurred in descending order of frequency: leucopenia (*n* = 16, 21.6%), neutropenia (*n* = 8,12.8%), anemia (*n* = 3, 4.1%), thrombocytopenia (*n* = 10, 13.5%), and hepatotoxicity (*n* = 2. 2.7%). 12 patients experienced rash, and 5 experienced fever. No serious gastrointestinal or renal toxicities were observed. Likewise, during the course of CCRT, the major acute grade 3–4 hematologic toxicities included leukopenia (*n* = 6, 8.1%), neutropenia (*n* = 9,12.2%), anemia (*n* = 3, 4.1%), thrombocytopenia (*n* = 5, 6.8%), and hepatotoxicity (*n* = 2. 2.7%). Grade 3–4 acute mucositis and dermatitis were reported in 5 (6.8%) and 2 (2.7%) patients, respectively.

**Table 5:** Acute side effects in 74 patients with locoregionally advanced nasopharyngeal carcinoma

Adverse events	During the period of IC					During the period of CCRT				
	0	1	2	3	4	0	1	2	3	4
Hematological										
Neutropenia	6	8	29	23	8	20	20	25	9	0
Leucopenia	9	9	40	15	1	26	20	22	6	0
Anemia	19	48	4	2	1	23	44	4	3	0
Thrombocytopenia	37	13	10	4	10	33	24	12	3	2
Non-hematological										
Hepatotoxicity	46	21	5	1	1	62	9	1	2	0
Nephrotoxicity	73	1	0	0	0	72	2	0	0	0
Mucositis	65	6	3	0	0	12	29	28	3	2
Dermatitis	74	0	0	0	0	0	54	18	2	0
Diarrhea	71	2	1	0	0	73	1	0	0	0
Nausea/vomiting	44	25	5	0	0	58	12	1	0	0

Abbreviations: IC induction chemotherapy, CCRT concurrent chemoradiotherapy.

## DISCUSSION

Since results of the 0099 trial showed that CCRT with or without AC yielded survival benefits over RT alone, CCRT has become a standard treatment for patients with locoregionally advanced NPC [14–16]. With the advent of IMRT, the local control rate increased to over than 90%, but distant metastasis still occurred in 15–20% of patients after IMRT plus CC [17]. A recent meta-analysis indicated that addition of chemotherapy to RT significantly improved survival outcomes of locoregionally advanced NPC patients [18]. So, IC or AC was an alternate modality added into the treatment of CCRT for these patients. A phase III randomized trial showed that addition of AC to cisplatin and fluorouracil (PF) after CCRT did not confer survival benefits to patients with locoregionally advanced NPC [6]. Thence, the addition of IC before CCRT seems to be an encouraging combined modality for locoregionally advanced NPC. However, PF-based IC followed by CCRT did not decrease metastatic failure in previous studies [18–20].

Some studies added intensive IC regimens to CCRT for patients with locoregionally advanced NPC. Several randomized phase 3 trials reported that the addition of taxane into the IC regimen of cisplatin with or without 5-fluorouracil (TPF or TP) improved the treatment outcomes in patients with locoregionally advanced head and neck squamous cell cancer [21–23]. Taxane-containing IC regimes provided equal survival benefits for patients with locoregionally advanced NPC [8, 9, 24]. It remains uncertain whether taxane-based IC regimens are the best options because of the high incidence of hematologic toxicity.

A GP-based regimen conferred survival benefit for patients with recurrent or metastatic NPC [11]. It remains controversial whether gemcitabine-containing regimens also increased survival outcomes for patients with locoregionally advanced NPC. Some retrospective studies showed that a GP regimen administered before RT obtained favorable survival outcomes with tolerable toxicities [12, 13, 25–27] (Table 6). Yau et al. retrospectively reported that GP is a well-tolerated and effective regimen with the overall response rate of more than 90%, 3-year OS of 76%, and 3-year DFS of 63% [25]. He et al. also indicated that the 3-year OS rate of locoregionally advanced NPC was 87.7% after GP-based IC plus IMRT [26]. A retrospective study performed by Jamshed et al. showed that the 5-year OS rate was 71% and the incidence of acute grade 3 toxicity related to the GP regimen was only 4% [27]. However, a randomized phase 2/3 trial conducted by Tan et al. found that the combination of gemcitabine, carboplatin, and paclitaxel IC plus CCRT failed to prolong 3-year OS, DFS, or DMFS compared with CCRT alone [28].

**Table 6:** Comparison of efficacy and toxicities in previous studies

	Study				
	Yau et al [25]	He et al [26]	Jamshed et al [27]	Zheng et al [13]	Our study
No of pts	37	54	99	13	74
Year	2006	2012	2014	2015	2017
Stage	IVA-B	IIB-IVB	IIB-IVB	III-IVB	III-IVB
RT technique	RT^*^	IMRT	CRT	RT/IMRT	IMRT
OS	76% (3-year)	87.7% (3-year)	71% (5-year)	83.9% (5-year)	81.9% (4-year)
PFS	63% (3-year)	-	50% (5-year)	-	77% (4-year)
DMFS	76% (3-year)	86.6% (3-year)	-	92.3% (5-year)	79.8% (4-year)
≥3 Toxicity	52%	9%	4%	-	21.6%

^*^accelerated radiotherapy.

Our phase II study assessed the clinical efficacy and toxicity of GP-based IC before CCRT for locoregionally advanced NPC. The study showed promising clinical outcomes, with 4 years LRFS of 86.9%, 4 year RRFS of 90.6%, 4 year DMFS of 79.8%, 4 year PFS of 77.0%, and 4 year OS of 81.9%. We attained similar survival outcomes as seen in the historical data [12, 13, 25–27]. Furthermore, univariate analysis revealed that clinical stage and CCRT regimens were significant prognostic factors for OS, while clinical stage was significant prognostic factor for PFS. In multivariate analysis, N3 was a poorer prognostic factor for OS and PFS, T4 for OS, 1 cycle of IC for RRFS. Although 21.6% of patients experienced grade ≥ 3 hematologic toxicity and 6.8% experienced grade ≥ 3 radiotherapy-related oral mucositis, only 2 patients were observed with grade 3 dermatitis within the RT field. Compared with TPF regimen [8, 29]. GP regimen obtained similar survival outcomes and lower incidence of grade ≥ 3 hematologic toxicity.

We found that GP-based IC before CCRT is an effective and well-tolerated modality for locoregionally advanced NPC. However, our results should be regarded as preliminary due to a small sample size and short follow-up time.

## MATERIALS AND METHODS

### Patients and pretreatment

The patients enrolled in this study were hospitalized from January 2012 to January 2014 in the Department of Radiation Oncology, Zhejiang Cancer Hospital. Eligible patients met the following criteria: (i) Histologically confirmed NPC; (ii) Aged 18 to 70 years; (iii) Stage III/IVA-B at diagnosis (American Joint Committee on Cancer staging system, 7th edition); (v) Adequate bone marrow, liver, and renal function; (vi) No previous anti-cancer treatment.

The exclusion criteria were: (i) patients were 70 years or older; (ii) had received RT, chemotherapy or surgery for tumors; (iii) had distant metastases before treatment; (iv) pregnancy; (v) history of other malignancy; and (vi) severe comorbidities. The prospective randomized study was approved by the medical ethics committee of Zhejiang Cancer Hospital. All patients signed written informed consent before participating in this research.

Patients received a pretreatment evaluation including complete history, physical examination, hematology and biochemistry profiles, chest radiographs, sonography of the abdomen, bone scan, magnetic resonance (MR) imaging of nasopharynx and nasopharyngoscopy. All patients were staged according to 2010 AJCC staging system. Tumor histology was classified according to the World Health Organization classification.

### Treatment schemes

#### Radiation therapy

All patients underwent radical IMRT with simultaneous integrated boost technique using 6 MV photons 2–3 weeks after IC. The delineation of target volumes of NPC during the treatment of IMRT was as described previously [30]. Briefly, gross tumor volumes of primary tumor and metastatic lymph nodes were defined as GTVnx and GTVnd, which were delineated according to pre- and post-IC MR images, respectively. The clinical target volume of nasopharynx (CTVnx) was defined as GTVnx plus a 7 mm margin that encompassed the nasopharyngeal mucosa plus 5 mm submucosal volume. The high-risk clinical target volume (CTV1) included the entire nasopharyngeal cavity, the anterior one- to two-thirds of the clivus, the skull base, the pterygoid plates, the parapharyngeal space, the inferior sphenoid sinus, the posterior one-quarter to one-third of the nasal cavity, and the maxillary sinus and any lymph nodes in drainage pathways containing metastatic lymph nodes. The low-risk clinical target volume (CTV2) included levels IV and Vb without metastatic cervical lymph nodes.

The planning target volume (PTV) was constructed automatically based on each volume with an additional 3-mm margin in three dimensions to account for set-up variability. All of the PTVs, including PGTVnx, PTVnx, PTV1, and PTV2, were not delineated outside of the skin surface. Critical normal structures including the brainstem, spinal cord, parotid glands, optic nerves, chiasm, lens, eyeballs, temporal lobes, temporomandibular joints, mandible, and hypophysis were contoured and set as organs at risk (OARs) during optimization.

The prescribed radiation dose was 70 or 72 Gy to PGTVnx, 66-70 Gy to PGTVnd, 62-66 Gy to PTVnx, 60-63 Gy to PTV1, and 51-54 Gy to PTV2, delivered in 30 or 33 fractions. Radiation was delivered once daily, five fractions per week, over 6 - 6.5 weeks for IMRT planning. The dose to OAR was limited on the basis of the Radiation Therapy Oncology Group (RTOG) 0225 protocol.

### Chemotherapy regimens

All eligible patients were given one to three cycles of GP-based IC (gemcitabine 1,000 mg/m2/day on days 1 and 8, cisplatin 25 mg/m2/day on days 1–3) at intervals of three weeks. Moreover, the patients in this study underwent CC with cisplatin (80 mg /m^2^) divided into 3 days and received AC with FP (cisplatin 25 mg/m2/day on days 1–3, and 5-fluorouracil 500 mg/m2/day on days 1–3) or GP regimens within 3–4 weeks after RT.

### Patient evaluation and follow-up

Tumor response was assessed by MRI and nasopharynx fiberscope according to the Response Evaluation Criteria for Solid Tumors criteria at three time points: after the completion of IC, at the end of IMRT, and 3 months after radiation. Systemic chemotherapy adverse effects were graded using the National Cancer Institute Common Toxicity Criteria (NCI CTCAE, version 3.0). RT-induced toxicities were scored according to the Acute and Late Radiation Morbidity Scoring Criteria of the RTOG.

Subjects underwent weekly examinations for treatment response and toxicities during radiation therapy. Patients were followed-up every 3 months for the first 2 years; every 6 months from the third to the fifth year, and then annually. Each follow-up included careful examination of the nasopharynx and neck nodes by an experienced doctor, MRI scan of the nasopharynx, nasopharynx fiberscope, chest computed tomography radiograph, and ultrasound of abdomen were performed 3 months after the completion of RT and every 6–12 months thereafter. Additional examinations were performed when indicated to evaluate local relapse or distant metastasis.

### Statistical analysis

Survival curves were generated using the Kaplan-Meier method and were compared using log-rank tests. Multivariate analysis was performed using Cox regression models to identify significant prognostic factors. Hazard ratios (HRs) and 95% confidence intervals (CIs) were calculated for each prognostic factor. IBM SPSS Statistics version 19.0 was used for all data analysis. Descriptive statistics was used to analyze the patterns of treatment failure. A *P* < 0.05 was considered statistically significant. Survival time was calculated from the date of diagnosis to the most recent follow-up or to either the date of relapse (event-free, local recurrence-free, or distant metastasis-free) or death (OS). After recurrence or metastasis, patients were given salvage therapy as determined by their physicians.
